# Penile Cancer: Innovations in Ultrastructural and Vibrational Markers

**DOI:** 10.1021/acsomega.4c07293

**Published:** 2025-01-23

**Authors:** Joel Félix Silva Diniz-Filho, Ana Caroline Muniz Silva, Antônio Augusto Lima Teixeira, Bruna Larissa Nolêto Sousa, Ralph Santos-Oliveira, Gyl Eanes Barros Silva, Clenilton Costa dos Santos, Luciana Magalhães Rebelo Alencar

**Affiliations:** † Biophysics and Nanosystems Laboratory, Department of Physics, Federal University of Maranhão, São Luís, Maranhão 65080-805, Brazil; ‡ Immunofluorescence and Electron Microscopy Laboratory (LIME/HUUFMA), Department of Medicine, Federal University of Maranhão, São Luís, Maranhão 65080-805, Brazil; § Brazilian Nuclear Energy Commission, Institute of Nuclear Engineering, Laboratory of Nanoradiopharmacy and Synthesis of New Radiopharmaceuticals, Rio de Janeiro 21941906, Brazil; ∥ State University of Rio de Janeiro, Laboratory of Radiopharmacy and Nanoradiopharmaceuticals, Rio de Janeiro 21941906, Brazil

## Abstract

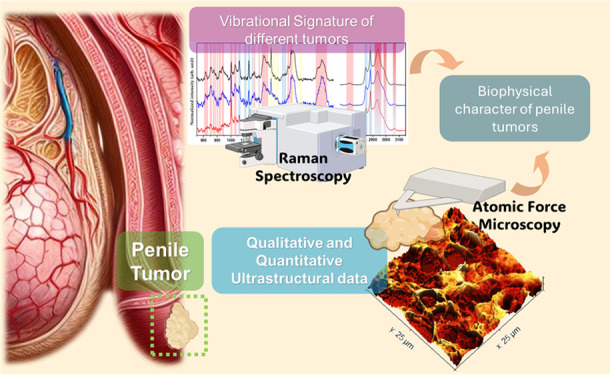

Penile cancer (PCa) is a disease that manifests predominantly as squamous cell carcinomas (SCCs), which, although rare, represents a significant public health problem, especially in regions with less socioeconomic development. One of the biggest challenges in managing this disease is the difficulty in differentiating tumor subtypes, making accurate diagnosis and treatment challenging. In this context, new characterization techniques are needed to investigate these tumors more completely. Atomic force microscopy (AFM) and Raman spectroscopy (RS) are valuable in this context, providing quantitative and qualitative ultrastructural data and vibrational signatures of the analyzed samples. In this study, AFM and RS techniques were employed to investigate subtypes of penile cancer, including the highly aggressive basaloid subtype, which is closely associated with human papillomavirus (HPV), and the sarcomatoid subtype, comparing them with nontumorous tissues. The AFM results revealed nanoscale changes in the ultrastructural properties of tumor samples, such as increased roughness in tumor tissues, with emphasis on the basaloid type associated with the HPV virus, and reduction in the surface area and volume of tumor tissues at the nanoscale, suggesting deeper tissue infiltration and greater deformability of tumor samples at the nanoscale. RS results detected significant spectral differences between normal and cancerous tissues and between tumor subtypes, particularly in vibrational modes related to proteins and lipids. Principal component analysis (PCA) confirmed a strong discriminative power between control and PCa groups. The data presented here offers new insights into the characteristics of penile tumors that, when integrated with clinical analyses, could improve the understanding of penile cancer behavior, contributing to more accurate diagnostic methods and targeted treatments.

## Introduction

Penile cancer (PCa) presents a significant geographic and socioeconomic disparity in its incidence rates. It is relatively rare in developed regions such as North America and Eastern Europe. In contrast, its prevalence is notably higher in developing countries across Asia, Africa, and South America.^1^ Recent data indicate a concerning uptrend in PCa incidence in these areas, highlighting the disease’s impact on public health and underscoring the need for targeted research and healthcare strategies to address this growing challenge.^2,3^

Histologically, most penile squamous cell carcinomas (SCCs) share morphological similarities with squamous neoplasms originating in other organs, particularly closely resembling those found in oral and cervical regions. This commonality underscores a shared pathogenesis that might be rooted in squamous cell pathology.^4^ About 70% of penile SCCs are classified as the usual type, characterized by their histological conformity to the classic presentation of squamous cell carcinomas. The remaining 30% of cases are distributed among distinct histological subtypes, including basaloid, verrucous, papillary, and sarcomatoid variants.^4−6^ Each of these histological subtypes exhibits unique clinical behaviors and prognostic implications.

Within the spectrum of penile squamous cell carcinoma (SCCs) subtypes, the basaloid and sarcomatoid variants merit specific focus due to their unique histopathological characteristics and clinical implications. Basaloid SCC, which represents about 10% of penile SCC occurrences, is more aggressive. This subtype is defined by the formation of small cellular clusters exhibiting minimal cytoplasm, pronounced nuclei, and a high frequency of mitotic figures, frequently in necrotic tissue. The designation “basaloid” reflects the morphological similarity of these neoplastic cells to basal cells. Histologically, basaloid SCC is identified by a distinctive architectural feature: a palisade-like configuration of cells at the periphery of tumor islands, coupled with a notable lack of intercellular bridges and the presence of central coagulative necrosis, often referred to as comedonecrosis. These defining characteristics contribute to the diagnostic criteria for basaloid SCC and underscore the subtype’s aggressive nature and potential impact on patient prognosis.^7^ Basaloid squamous cell carcinoma (SCC) is characterized by its intimate interaction with the surrounding connective stroma, which supports clusters of basaloid cells. This interaction manifests as distinctive slits or interfaces, commonly referred to as the epithelium-stroma interface. These morphological features facilitate the carcinoma’s identification and aggressive behavior and frequently demonstrate a propensity for deep infiltration into the underlying tissues. A significant clinical concern with basaloid SCC is its high rate of lymphatic spread; more than half of the patients exhibit involvement of the inguinal lymph nodes at the time of initial diagnosis. This pattern of aggressive growth and early lymphatic involvement underscores the importance of prompt, accurate diagnosis and comprehensive management strategies to address both the primary tumor and potential metastatic disease.^8^ This subtype of PaC is directly associated with HPV.

Human papillomavirus (HPV) represents a pivotal factor in the etiology of penile cancer, a malignancy that, although relatively uncommon globally, poses significant health challenges in various regions, especially in developing countries. HPV, a DNA virus from the Papillomaviridae family, is known for infecting epithelial cells of the skin and mucous membranes, leading to a range of outcomes from benign lesions to malignancies.^9,10^ The oncogenic potential of HPV, particularly high-risk subtypes such as HPV 16 and 18, is linked to their genetic material integrating into the host cell’s DNA, thereby disrupting normal cell cycle regulation and promoting malignant transformation.^11,12^ This process is mediated by the viral oncoproteins E6 and E7, which interfere with tumor suppressor proteins p53 and retinoblastoma (Rb).^13^

Although rare (occurring in less than 1% of cases), the sarcomatoid subtype demonstrates significant biological aggressiveness, attaining considerable sizes and penetrating deeply into adjacent structures. Microscopically, it is characterized by spindle-shaped cells intermingled with cells of bizarre or giant shapes and may present sarcomatous components, such as chondrosarcoma or osteosarcoma. The histology of sarcomatoid SCC is biphasic, involving the differentiation of squamous epithelial and mesenchymal components. In the transformation process from squamous cells to spindle cells, the epitheliomesenchymal transition occurs through a decrease in the expression of E-cadherin, the primary epithelial intercellular adhesion molecule, and an increase in the expression of N-cadherin, responsible for the mobile phenotype of the cells.^14^

Atomic Force Microscopy (AFM), a scanning probe microscopy technique developed by Gerd Binnig and collaborators,^15^ is an important tool in nanoscale investigation, enabling measurement between the probe and the surface sample atoms, with resolution on the order of pN. One of the most significant advantages of AFM is the ability to analyze a wide range of samples (not just conductive ones), including tissues, cells, and viruses.^16,17^ AFM has been frequently used in tumor tissue and cell research.^18^ The use of AFM as a quantitative biomarker for cancer-related changes has been demonstrated for various types of cancer, including breast,^19,20^ prostate,^21^ ovarian,^22^ pancreatic,^23^ kidney^20^, and bladder cancers.^24^

Raman Spectroscopy (RS), developed by Chandrasekhar V. Raman and Krishnan,^25^ is a technique based on the incidence of a beam of monochromatic light on a sample, taking into account the light scattered in an inelastic way (Raman scattering) from the sample surface. Raman scattering provides important information about the samples’ molecular vibrations and chemical bonds under study.^26,27^ In the field of cancer diagnosis, various research groups have explored the feasibility of RS for diagnosing cancer in different organs, such as the colon,^28^ cervix,^29^ esophagus,^30^ stomach,^31^ mouth,^32^ and skin.^33^ In the study of penile cancer, RS can be particularly valuable, especially in identifying vibrational modes that may be associated with HPV (Human Papillomavirus) infection, which is common in penile tissue affected by tumors.^34^

The combination of AFM and RS in studying cancerous tissues and cells has shown promising results, providing detailed information about nanostructures and their functional alterations. Compared to nontumor samples, cancerous cells and tissues exhibit irregular morphology, altered deformability,^35^ and altered molecular characteristics, such as genetic mutations and dysregulation of signaling pathways.^36^

The limited evidence on penile squamous cell carcinoma with epithelioid features (PCa) underscores a critical gap in our comprehensive understanding of its tumor biology. To bridge this knowledge gap, this study integrates Atomic Force Microscopy (AFM) and Raman Vibrational Spectroscopy (RS) as investigative tools for a detailed ultrastructural and molecular examination of PCa tumors, specifically focusing on sarcomatoid and basaloid subtypes. The juxtaposition of ultrastructural and molecular data from these advanced imaging techniques promises to shed light on the complex biology of penile squamous cell carcinoma, potentially paving the way for new approaches to its unique pathological features.

## Methodology

### Tissue Selection

Specialized researchers collected the samples from three reference hospitals located in São Luís, Maranhão (Presidente Dutra University Hospital, Aldenora Bello Cancer Hospital, and Maranhão Cancer Hospital Dr. Tarquínio Lopes Filho). Participants were informed about the study’s research objectives, risks, and expected impacts. All participants signed the Informed Consent Form (ICF), which the Human Research Ethics Committee approved. Subjects who agreed to participate in the research were interviewed for socio-behavioral data collection using a data collection instrument. In contrast, those who did not agree were assured that there would be no harm to conventional hospital treatment and follow-up. After the material was collected for research, all materials were identified using a specific project code to ensure participants’ confidentiality and privacy rights.

### Inclusion Criteria

This study considered men over 18 years of age with clinical and anatomopathological diagnoses of penile cancer who had an amputation as the first therapeutic option. Only those who agreed to participate in the study by signing the Informed Consent Form (ICF) were included.

### Exclusion Criteria

Those who had undergone chemotherapy or radiotherapy before the surgical procedure were excluded.

### Review of Histological Slides and Selection of Study Area

After applying inclusion and exclusion criteria, cases were selected, and their Hematoxylin and Eosin (H&E) stained slides underwent a reevaluation to confirm histological diagnosis and tumor classification according to the criteria proposed by the American Joint Committee on Cancer (AJCC).^37^ The slide review was conducted independently by two pathologists. Tumor subclassification (SCCs) was based on criteria established in the medical literature.^38^

### Tissue Collection

The samples used in this study were collected in the surgical center: the physician responsible for the amputation collected small fragments of fresh tissue containing tumor and nontumor (normal) samples. The samples were stored in the following solutions: (1) RNAlater (ThermoFisher) for DNA extraction and HPV detection and (2) 10% buffered formalin for biophysical analyses.

### HPV Detection and Genotyping

The QIAamp Fast DNA Tissue kit (Qiagen, Cat. No. 51404) was used for DNA extraction. The extracted samples were evaluated for extraction quality by quantifying the total material on a NanoDrop spectrophotometer (ThermoFisher), with concentrations expressed in ng/μL, and purity assessment with 260/280 nm measurements (between 1.8 and 2.0) and 260/230 (above one). The samples were stored at −20 °C until used in subsequent steps.

HPV detection was conducted by conventional PCR (Polymerase Chain Reaction) in two stages (nested PCR). In the first PCR, a set of generic primers called PGMY09/11, described by Gravitt et al.,^39^ which produced a 450 bp fragment of the HPV capsid L1 region, was used. The primer GP5+/6+ was used in the second PCR, generating a 170 bp amplicon corresponding to the viral capsid L1 region. A pair of primers for the β-globin gene (366 bp fragment) was used as a positive control for the reaction. The final mix was 25 μL for each sample, using the MASTERMIX PCR PLATINUM SUPERFI kit (Life Technologies), followed by 45 cycles of 94 °C for 45 s, 40 °C for 1 min, and 72 °C for 1 min, and 72 °C for 10 min. The amplicons were separated on a 1.5% agarose gel and subjected to a constant voltage of 90 V for 40 min. Only those with amplification for the β-globin and GP5+/6+ genes were considered positive. Capillary electrophoresis sequenced positive cases, and their sequences were compared to those available in genetic databases using BLAST (Basic Local Alignment Search Tool) for viral genotyping.

### Tissue Preparation

The PCa samples will be embedded in paraffin and cut using an ultramicrotome (model LEICA EM UC6), producing 2 μm thick sections. The biopsies will be deposited on 13 mm diameter glass slides and subsequently placed in an oven at 60 degrees for 30 min for dewaxing. After this process, the samples will be submerged in 30 mL of Xylene and gently shaken for 15 min, with 30 mL of Xylene being changed every 5 min. Subsequently, the samples will be rehydrated through a sequence of 90, 80, and 70% PA ethyl alcohol for tissue rehydration.

### Atomic Force Microscopy Setup

The analysis by Atomic Force Microscopy was conducted using an AFM Multimode 8 (Bruker, Santa Barbara, CA, USA) in PeakForce Quantitative Nanomechanics mode – QNM. For this purpose, probes of the qp-HBC model (NanoSensors) with a nominal cantilever spring constant of 0.5 N/m and a tip radius smaller than 10 nm were utilized. All data were obtained with a scanning rate of 0.5 Hz and a curve acquisition frequency of 0.5 kHz. Three nontumorous samples (Control Group), three samples of the sarcomatoid subtype, and three samples of the basaloid subtype were used. Each sample underwent 15 scans at distinct points. In total, 45 maps of 25 μm × 25 μm were analyzed for each group. Each scan contained 65536 force curves, providing a broad database for comparative analysis among the studied groups.

### Ultrastructural Analysis

The roughness was calculated using statistical methods based on the height data of each pixel in the image, derived from the height map. The root-mean-square (RMS) roughness *R_q_* is defined by eq 1:
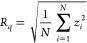
1where *z* is the height of each pixel, and *N* is the total number of pixels in the image.^40^ In this analysis, images were scanned at 25 μm resolution. A third-order polynomial fit was applied to minimize height differences, ensuring that tissue structures were accurately represented.^41^

The projected surface area was calculated by simple triangulation, dividing the surface into small triangles formed by the pixels using statistical analysis from the software Gwyddion 2.60.^42^ The volume was obtained by integrating the surface height over the covered area and assessing topographic variations. The deformability was evaluated using the area-to-volume ratio, which influences the performance and stability of cells. According to Amorim et al., the relationship between surface area *a* and volume *v* can be employed to define membrane deformation.^43^

### Raman Spectroscopy Analysis

Raman Spectroscopy was used to analyze and identify the spectral differences obtained from the control group and groups of patients with PCa, previously disclosed through the clinical method. The measurements were carried out on Horiba’s T64000 spectrometer with a CCD (Charge Coupled Device) detection system cooled with liquid nitrogen. All measurements were obtained in backscatter geometry. The 532 nm line was used as a traction source with its maximum power for the measurements. The sample surface was viewed using a specific Olympus brand with an attached video camera. To focus the brightness on the surface, we used a 100× lens. Nine acquisitions were carried out with times of 20 s. The spectral region observed in our experiments was divided into intervals of 750 to 1750 cm^–1^ (Low Wavenumbers – LWN) and 2650–3150 cm^–1^ (High Wavenumbers – HWN)

### Spectral Preprocessing

Data processing was conducted using LabSpec6 software. The narrow peaks caused by cosmic rays were sequentially removed, and the variable fluorescence background and the glass substrate were estimated using a fifth-order polynomial fitting and subsequently subtracted. Each spectrum was smoothed using a polynomial smoothing algorithm before analysis.

### Principal Component Analysis

Principal Component Analysis (PCA) was applied to the spectral data set, a statistical analysis method capable of reducing the dimensionality of the data while capturing most of the variation in the original data set. The spectra were analyzed following the methodology of Yi Hong Ong and co-workers,^44^ where variance analysis was employed on the scores of the first ten principal components to determine which PC exhibited significant differences in mean scores between the two groups of cells, utilizing OriginLab software.

### Statistical Analysis

Statistical test following a single criterion was evaluated using ANOVA and Tukey’s post-test, considering the values were statistically significant when *p* < 0.05. Statistical analyses and graphics were performed using the ORIGIN software. The calculated error was the standard deviation (SD) in all data.

## Results

**Figure 1:**
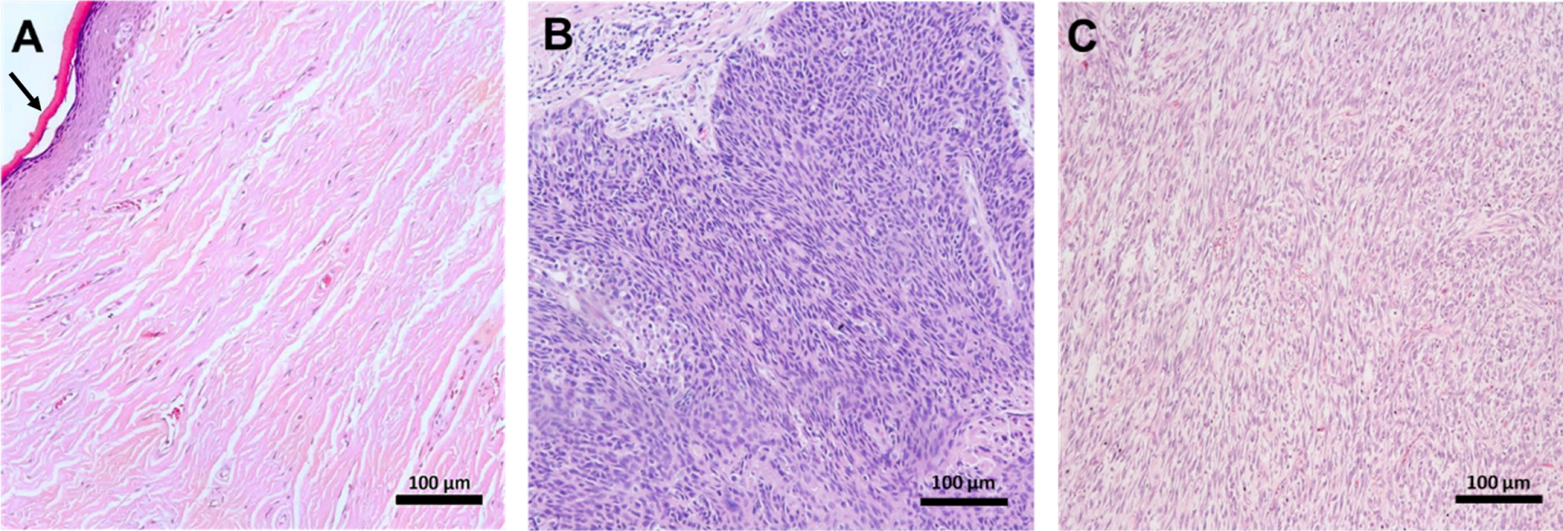
Optical microscopy. Representative optical microscopy images of tissues from the control group (nontumorigenic) (A) and those with PCa: basaloid (B) and sarcomatoid (C). The black arrow indicates the outermost layer, the stratum corneum.

Figure 1 shows a representative optical microscopy image of each tissue analyzed. In Figure 1A, representing the control group, the histological slide shows the layers of the epidermis with stratified squamous cells, without neoplastic changes, with the stratum corneum being the outermost layer with a pink color, as shown by the arrow, and the basal layer being the innermost with large, elongated and hyperchromatic nuclei pigmented with hematoxylin.^45^Figure 1B shows the basaloid group, indicated by the abnormal growth of bluish, small, uniform cells with round nuclei and scant cytoplasm, which resemble the basal cells of epithelial tissue, normally presenting a palisade arrangement in the peripheral cells of the tumor islets, absence of intercellular bridges and the presence of central coagulation necrosis.^46,47^Figure 1C represents the sarcomatoid group, marked by the differentiation of squamous and mesenchymal components, characterized by the expression of spindle-shaped sarcomatous cells, which exhibit atypical and elongated nuclei, resulting from the epitheliomesenchymal transition, in which the squamous cell is transformed into spindle cells.^48^ Such histopathological components show the conformational changes undergone by the pathological tissue compared to the control group.

**Figure 2:**
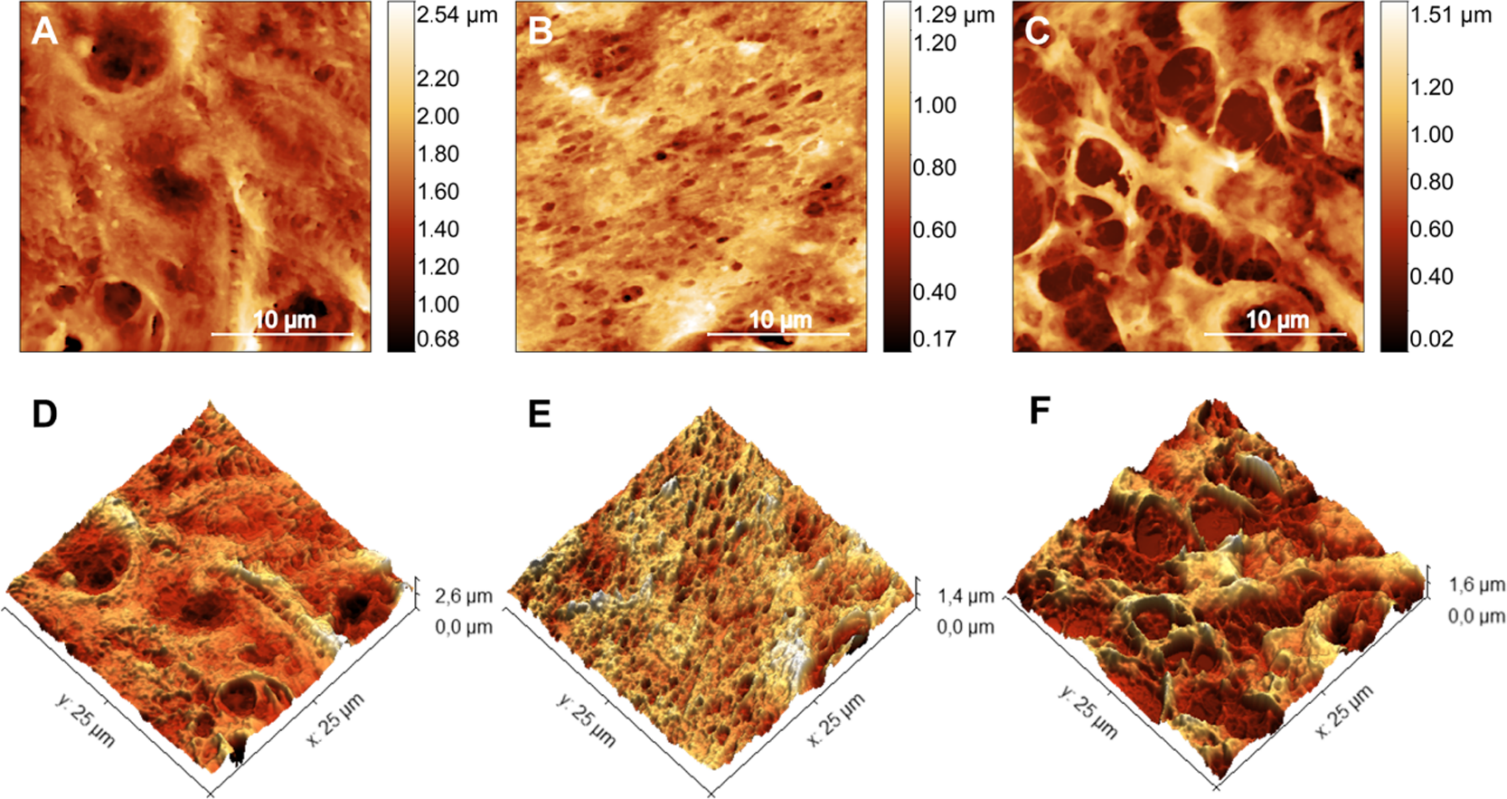
Atomic force microscopy maps. 25 × 25 μm topographic AFM maps of the ultrastructure of tissues from the control group (nontumorigenic) (A) and those with PCa: basaloid (B) and sarcomatoid (C) and its respective three-dimensional representations (D–F).

The representative high-resolution AFM maps of each group reveal ultrastructural changes on the surface of PCa tissues, as observed in Figure 2. Figure 2A shows a 25 μm^2^ scan over a nontumor region of penile tissue and its respective 3D view in Figure 2D, compatible with the preserved ultrastructural morphology of the stratum spinosum layer.^49^ In contrast, Figure 2B (Figure 2E, 3D view) shows a scan of the same size for basaloid cancer tumor tissue associated with HPV infection.^50^ The submicrometric-sized holes in Figure 2B may be related to the percolation or diffusion of viral particles in this type of tumor. In PCa sarcomatoid subtype tissue, as shown in Figure 2C (Figure 2F, 3D view), several stretches of tissue form micrometric holes on its surface. This fact is associated with high vascularity in cancer tissues, indicating rapid tumor growth, as vascularization is necessary to supply nutrients and oxygen to cancer cells.^51^ It is important to highlight that the structural changes observed in each sample group are related to the pathogenic process of tumor progression in the cancerous groups and are not correlated with artifacts introduced during the sample preparation process or the scanning procedure using Atomic Force Microscopy. Despite the possible changes in tissues that the preparation process can cause, many studies reveal how reliable this process is^52−56^ in studying the characteristics of different tissues, confirming that the main changes observed in various types of tumors here investigated are associated with cancer and how it is responsible for altering tissue architecture. Furthermore, all sample groups, including the control and tumor groups, underwent the same preparation process, ensuring that any observed differences are inherent to the biological condition of the tissue and not a result of sample handling.

**Figure 3:**
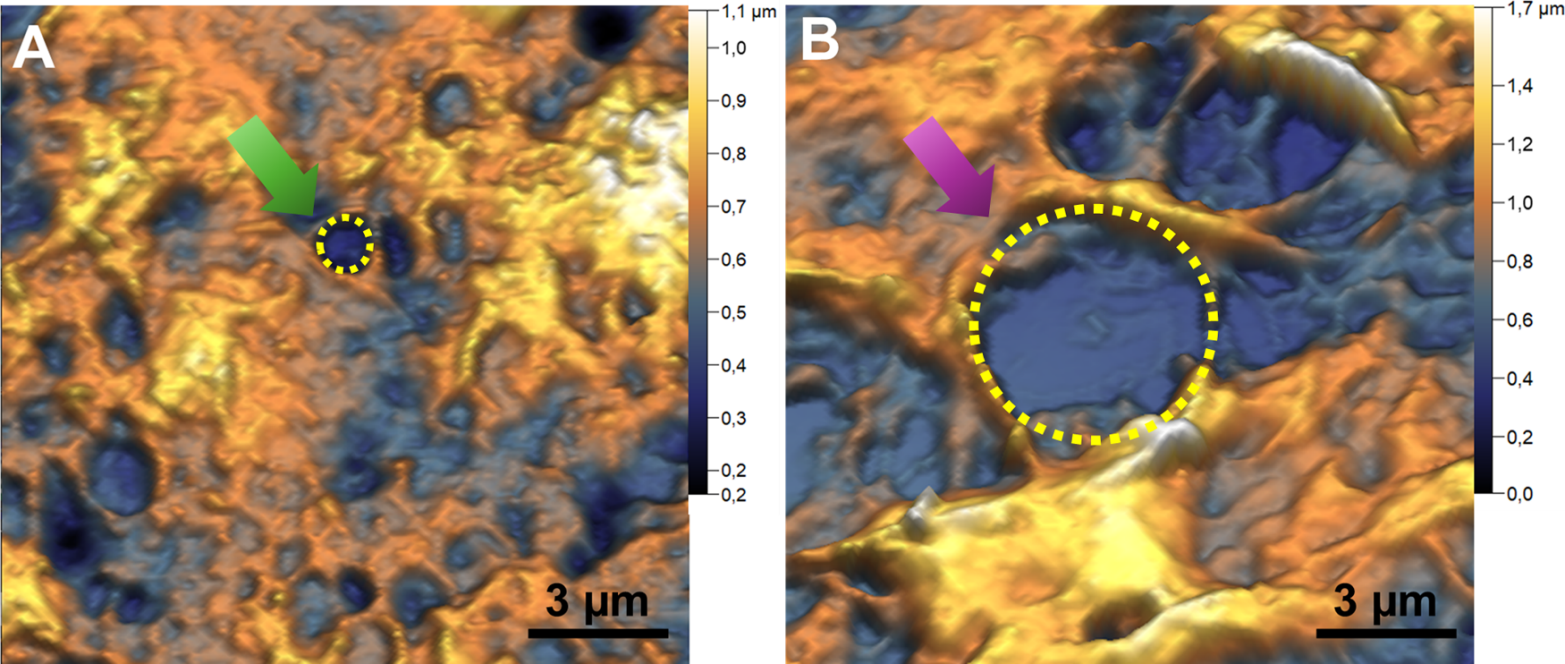
PCa surface porous. Topographic AFM maps of the ultrastructure of tissues from basaloid (A) and sarcomatoid (B) tumors. Arrows point to the holes observed in each cancer subtype, and dotted circles delimit the holes in each tumor type.

In Figure 3, one can see how each PCa subtype promotes fenestrations in the tumor tissue. Figure 3A shows a representative image of the basaloid subtype, showing uniform fenestrations (or pores) of submicron diameter (0.69 ± 0.05) μm. As with many medications,^57−60^ which prevent or reduce its effectiveness, viral particles can become trapped by this complex porous structure, which may associate this subtype with HPV infection. Figure 3B shows a representative image of the surface detail of the sarcomatoid subtype tumor tissue. Here, it is possible to observe a greater presence of dark regions (holes), micrometric in size (6 ± 1) μm, compatible with tissue failures. These holes have a medium diameter compatible with capillaries that irrigate the tumor tissue.^61,62^ When the vascular and nutritional supply aligned with the high mitotic activity of the tumors does not supply the demands of the tumor microenvironment, the formation of foci of necrosis, typical in basaloid and sarcomatoid PCa, occurs, visible through the stretches.^63^ Furthermore, high vascularity can also facilitate the spread of cancer to other parts of the body through the bloodstream.

**Figure 4:**
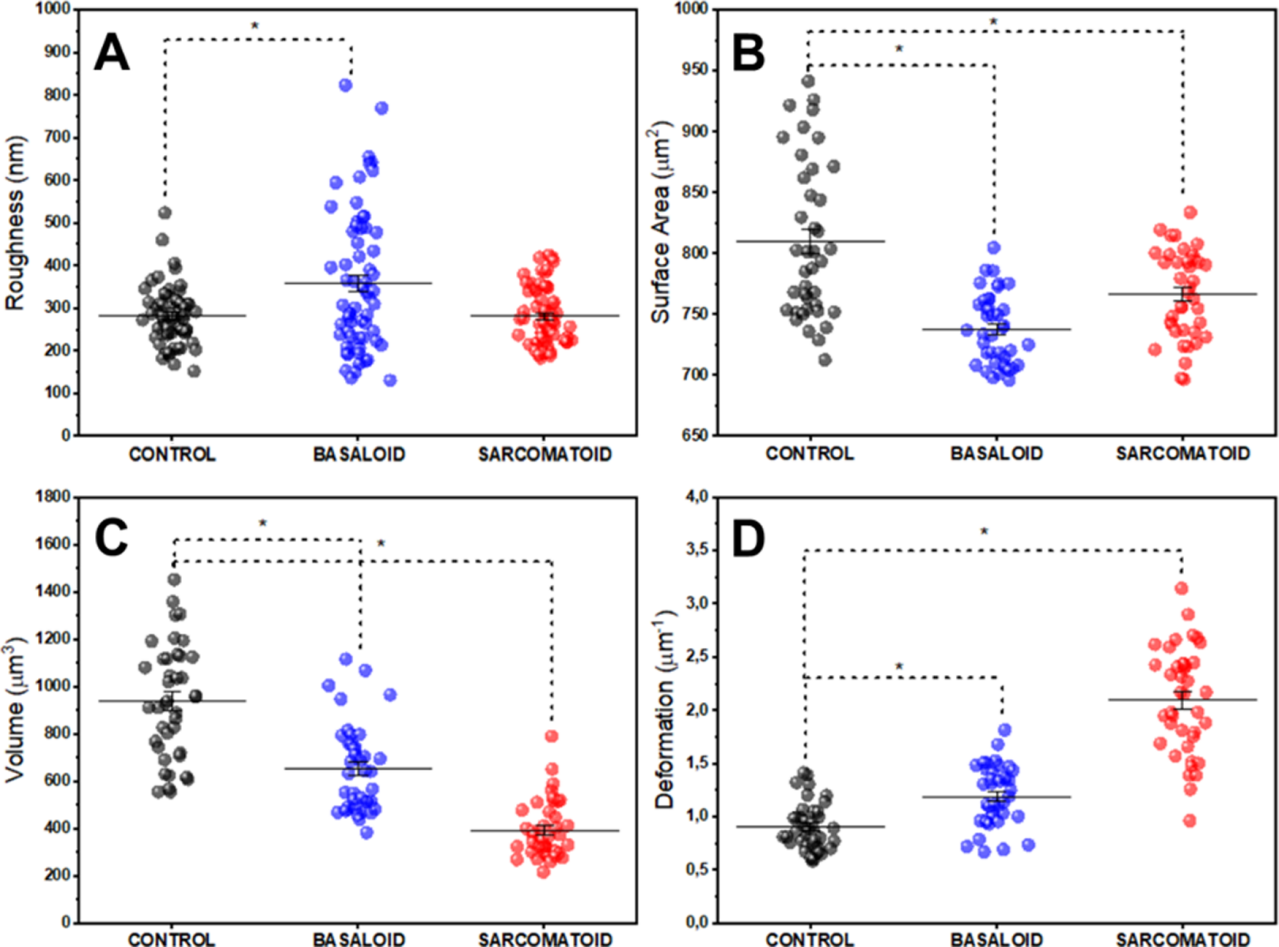
AFM quantitative data. Quantitative data of ultrastructural properties of tissues from the control group (nontumorigenic) and PCa patients. (A) Roughness, (B) tissue surface area, (C) volume, and (D) deformation scattering charts. The (*) indicates significant differences in the ANOVA test with Tukey for *p* < 0.05.

Motivated by these qualitative ultrastructural differences observed in tumor tissues concerning nontumor tissue and also between the different types of tumors (basaloid and sarcomatoid), we analyzed quantitative ultrastructural parameters of the groups, such as mean quadratic roughness of the tissue surface, surface area, tissue image volume and deformation (*A*/*V* ratio). The results can be seen in the panel shown in Figure 4. The scatterplot shown in Figure 4A presents the mean squared roughness results for each group analyzed. The mean values and their respective standard deviations are (284 ± 8), (360 ± 10), and (284 ± 8) nm for the control, basaloid, and sarcomatoid tissues. A greater number of holes (fenestrations) in the basaloid tumor tissue is reflected in the increased roughness result, which may be associated with the greater capacity of these tumors to trap a greater quantity of viral particles.^13^

Figure 4B shows the scatter plot of surface area values of tissues from each group analyzed. The average values obtained were (810 ± 10), (738 ± 5), and (767 ± 6) μm^2^, respectively for the control, basaloid and sarcomatoid groups. Here, it is possible to observe a trend, with statistical relevance, of a decrease in the surface area of tumor tissues when compared with nontumor ones.

Figure 4C presents the volume results of the maps obtained from the analyzed tissues. The average values obtained were (940 ± 40), (660 ± 30), and (390 ± 20) μm^3^ for the control, basaloid and sarcomatoid groups, respectively. As with the surface area, the maps obtained from the tumor tissue samples showed a reduced average volume compared to the control group.

The graph shown in Figure 4D shows the scatter plot of surface deformation calculated from the geometric parameters of the images obtained from each group. The average values found were, respectively, (0.91 ± 0.03), (1.20 ± 0.04), and (2.10 ± 0.07) μm^–1^ for the control, basaloid and sarcomatoid groups. It is possible to observe greater deformability in tumor samples than in nontumor ones. This fact may be associated with the greater capacity for tumor deformation at the cellular level,^64,65^ since we are analyzing the ultrastructure of tumor tissues, which enables greater invasion of these tumors into new sites.

**Figure 5:**
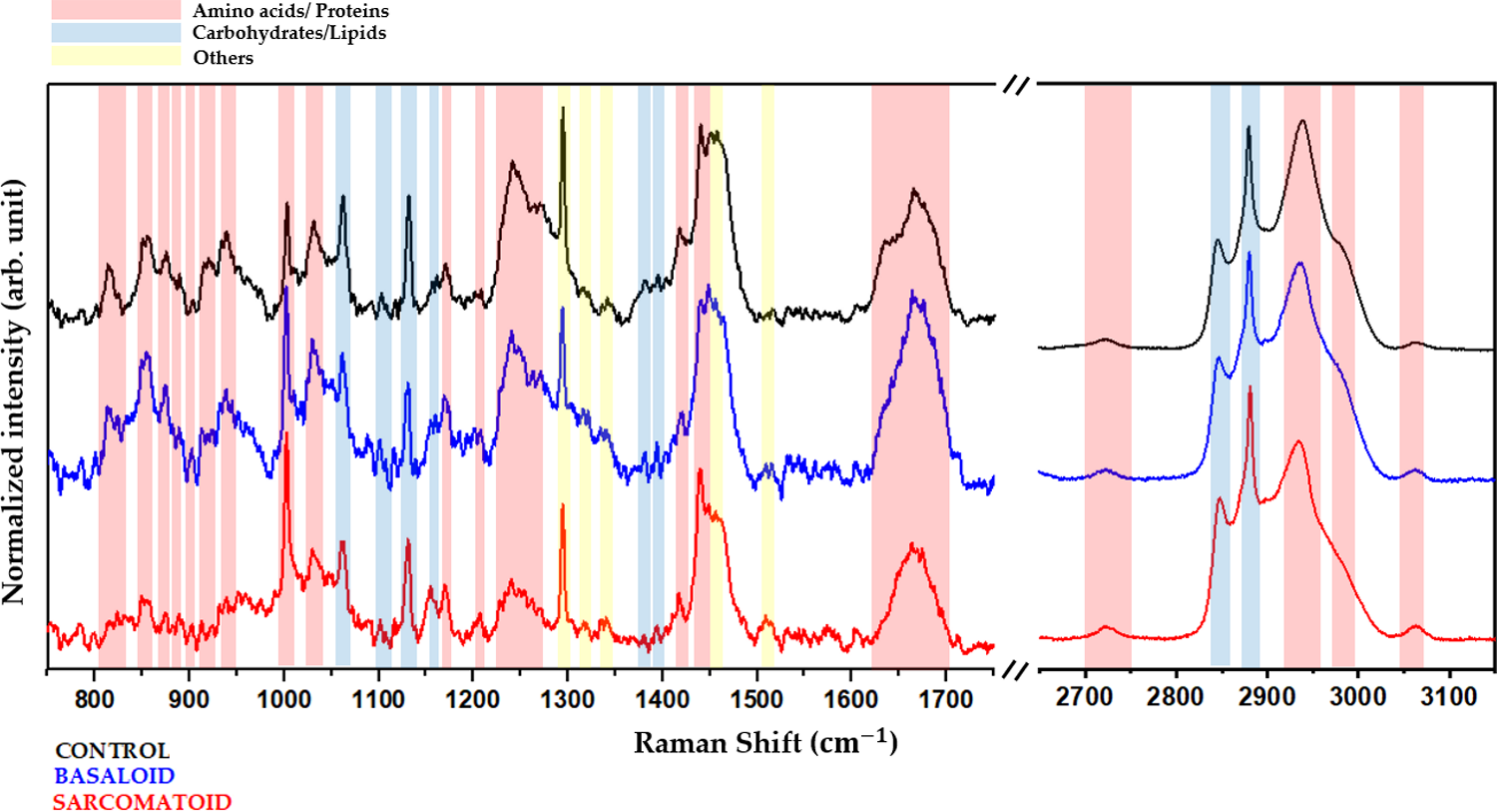
Molecular identification. Average spectra and identification of vibrational modes related to the control group (black), sarcomatoid group (red), and basaloid group (blue). The red strips identify the bands corresponding to proteins and amino acids, the blue strips correspond to the lipid and carbohydrate modes, and the yellow strips correspond to other modes.

Another promising approach is investigating the vibrational signature obtained through Raman Spectroscopy in the tissues examined. This biochemical study, combined with AFM data, may bring new perspectives to the study of penile tumors. Figure 5 shows the average spectrum obtained from 30 samples from each group. The bands refer to the vibrational modes associated with the main biochemical components of tissues, such as proteins and amino acids, carbohydrates, and lipids, among others, for both low and high wavenumber. This way, it is possible to observe the differences in the biochemical composition among the tissues analyzed.

The specific wavenumber for each identified mode can be found in Table 1.

**Table 1:** Assignments of Each Mode of the Tissue Raman Spectrum^27,67−70^^a^

wavenumber(cm^–1^)	amino acid/protein	lipid/carbohydrate	other
813	C–C str.		
856	proline		
874	C–C str.		
888	protein		
919	proline		
937	proline		
1002	phenylalanine		
1031	phenylalanine		
1058		lipids	
1100		lipids	fatty acid
1131		phospholipids	
1159	C–C/C–N str.		
1169	proline		
1207	hydroxyproline, tyrosine		
1239–1272	amide III		
1293			cytosine
1315			guanine
1340			nucleic acid
1381		δCH_3_	
1392	C–N str.		
1402	methyl ben groups		
1416	C=C str.		
1450	CH_2_ ben.		
1458			nucleic acid
1514			cytosine
1638–1665	amide I		
2728	C–H str.		
2853		CH_2_ sym. str.	
2888		CH_2_ asym. str.	
2935	CH_3_ sym. str.	CH_3_ sym. str.	
2960–2980	CH_3_ asym. str.	CH_3_ asym. str.	
3008		=CH str.	
3030	aromatic	aromatic	
			

a Abbreviations: str. = stretching, sym. = symmetric, asym. = asymmetric, def. = deformation, ben. = bending.

**Figure 6:**
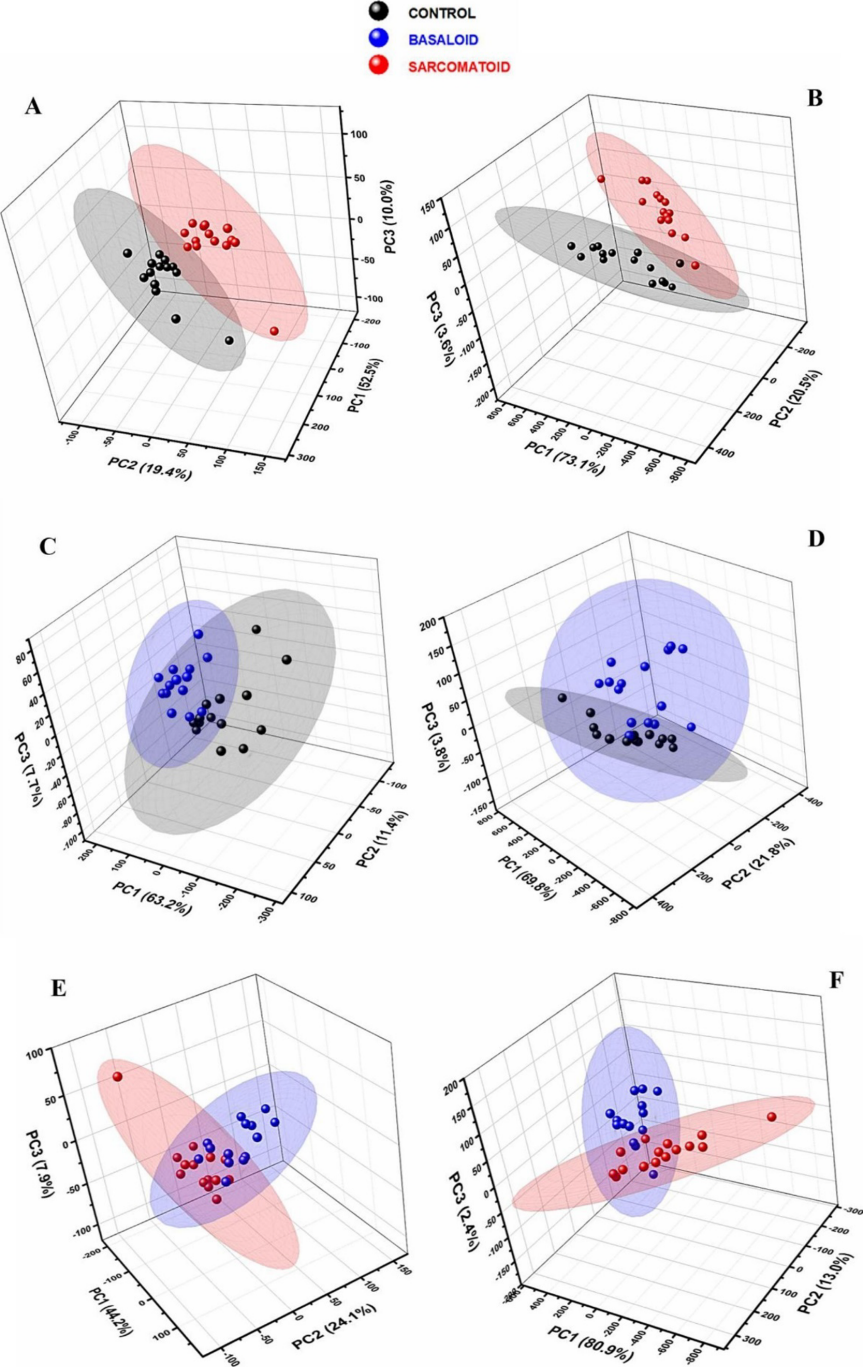
PCA analysis from Raman spectra. (A) Control × sarcomatoid in LWN; (B) control × sarcomatoid in HWN; (C) control × basaloid in LWN; (D) control × basaloid in HWN; (E) sarcomatoid × basaloid in LWN; and (F) sarcomatoid × basaloid in HWN.

To evaluate the discriminatory capacity of the method used through multivariate analysis, Principal Component Analysis (PCA) was performed on all data contained in the Low Wavenumber (LWN) and High Wavenumber (HWN) regions. In total, 30 spectra from each sample group were analyzed, suitable for statistical analysis. The ellipses present in the graph delimit the area in which 95% of the data is included. In Figure 6, the first three main components are highlighted, which result in good total variability of the data set.

Principal components (PCs) are new variables generated by PCA that summarize the variance in a multidimensional data set. PC1 captures the largest portion of the variation, followed by PC2 and PC3, with each component uncorrelated with the previous one. PCA helps reduce the complexity of the data, making it easier to identify important patterns and highlight the most relevant differences between the analyzed groups.^66^

Figure 6A shows the confidence ellipses for the control and sarcomatoid groups considering the spectrum region between 750 and 1750 cm^–1^ (low wavenumbers – LWN). The first three main components add up to 81.9% confidence. Figure 6B shows the relationship between the same groups, now for high wavenumbers (HWN), between 2650 and 3150 cm^–1^. In this spectrum range, the first three PCs total 97.2% confidence. Figure 6C shows the confidence ellipses for the control and basaloid groups for LWN, with a confidence of 82.3% for the first three PCs. In contrast, Figure 6D shows the relationship between the same groups for HWN, with a confidence of 95.4% for the first three PCs. Figure 6E,F shows the relationship between the tumor groups (basaloid and sarcomatoid) for LWN (76.2%) and HWN (96.3%), respectively.

## Discussion

The results suggest ultrastructural modifications in the morphology of tumor tissue. Sarcomatoid PCa has a biphasic character, marked by a squamous component with sarcomatous differentiation of spindle cells.^14^ This epitheliomesenchymal transition is characterized by decreased expression of E-cadherin and increased expression of N-cadherin, which is responsible for the mobile phenotype of the cells.^14^ Cadherins are polypeptides responsible for epithelial intercellular adhesion, associated with a group of catenin proteins that bind the actinic microfilaments of the cytoskeleton.^71^

Zemła and co-workers demonstrated with AFM that the most rigid conformations within the cell surface are made up of actin filaments, and the structural disarrangements in the organization of the cytoskeleton were attributed to lower cellular rigidity, giving a mobile aspect to cancer, which correlates with the modulations caused by losses of E-actin-bound cadherins. Changes in cytoskeletal dynamics, mediated by changes in cadherin expression, can influence cell morphology, indicated by changes in area and volume, which express a reduction in volume and area data in the sarcomatoid group.^72^ AFM results (especially topography and deformation) show nanoscale changes in tumor tissues compared to the control sample. This relationship between increased deformation and nanoscale structural changes is strongly linked to membrane changes and, mainly, in the composition and arrangement of the cytoskeleton.^35^

Another possible explanation to describe the structural and functional changes of the actin cytoskeleton is L-plastin, a group of actin-bridging proteins that contribute to tumor cell invasion in a phosphorylation-dependent manner.^73^ Phosphorylation of L-plastin at its Ser5 residue increases its ability to interact with actin, thus influencing its intracellular localization.^73^ The supply of energy to trigger phosphorylation on the L-plastin residue may be associated with glycolytic enzymes, which in cancer cells, due to the high rate of glycolysis, are increased, producing ATP in the vicinity of the cytoskeleton through reversible binding of glycolytic enzymes to the cytoskeleton.^74^

PCa has subtypes associated with HPV, such as basaloid, and nonassociated subtypes, such as sarcomatoid.^2^ HPV-related penile carcinogenesis, typical of the basaloid subtype, arises from the overexpression of the viral oncoproteins E6 and E7, causing cell cycle dysregulation and genomic instability.^13^ The viral oncoprotein E6 interferes with the p53 pathway, a tumor suppressor protein, inhibiting apoptosis by targeting the protein for degradation. The inhibition of p53 by E6 promotes exacerbated cell proliferation and tumor cell immortalization. However, non-HPV-associated carcinogenesis, such as in the sarcomatoid subtype, may result from mutagenic changes in tumor suppressor genes.^75^ In a study carried out by Jacob et al., patients with metastatic penile cancer had mutations in TP53.^27^ HPV-negative results suggest that mutations in the TP53 gene, leading to the overexpression of p53, are linked to cancer metastasis and poorer survival outcomes in patients with advanced stages of the disease.^2^ The p53 pathway is a regulator in the formation of tumor-associated collagen signature, a collagen bundle angled at 60° to 90° to the edge of the cancer, and is indicated by cancer proliferation and invasion.^76^ The changes show that the sarcomatoid subtype presents a specificity in the expression of collagen in the extracellular matrix that can alter its ultrastructural properties, such as the reduction in area and volume, associated with ECM modulations that favor the formation of apertures.

The modification in surface roughness may be associated with biological processes underlying cancer development, such as uncontrolled cell proliferation and reorganization of the ECM.^74^ Metastatic cancer cells exhibit an expanded expression of transport proteins such as ion channels, ion transporters, and aquaporins. These ion/water transport proteins, such as NHE1, NKCC1, AE2, ENaC, AQPs, IK channel, VRACs, ClC-3, and TMEM16s, often demonstrate elevated activity or expression in cancer cells. The increase in expression of these membrane proteins may justify the roughness observed in basaloid and sarcomatoid PCa, indicating a possible adaptation of cancerous tissues for more effective and invasive dissemination in other tissues.^77^

There was a reduction in the surface area of PCa tissues, which was more evident in the basaloid subtype, affected by the HPV virus. When comparing the samples with the control group, a similar pattern can be seen in the maps, with a decrease in height between the sarcomatoid and basaloid samples. This reduction in surface area suggests greater aggressiveness of the tumor, especially in the basaloid and sarcomatoid subtypes, classified as aggressive and with a high rate of nodal metastasis.^78,79^

Compared to control samples, the analysis of basaloid and sarcomatoid samples revealed a reduction in volume. Transport proteins, such as ion channels, ion transporters, and aquaporins (AQPs), regulate cell volume during exposure to osmotic stress.^77^ Studies indicate that water flow related to osmotic gradients generated by ionic transport contributes to cell migration.^80−83^ It was reported that cell migration is attenuated by extracellular hypertonicity; cell shrinkage, which inhibits local volume, would facilitate cell migration.^82^ Furthermore, the osmotic gradient is responsible for regulating the expression of ion/water transport proteins and their changes in location in the membrane, modulating the cycles of protrusion of the leading edge and retraction of the rear part of the cell during migration.^84^ These aspects demonstrate that changes in protein expression in metastatic cells, altered by extracellular osmotic stress, directly impact cell migration, typical of metastatic cancer. The correlation with volume and deformation data shows that changes in cellular structure possibly caused by the osmolarity of the medium can impact the reduction of metastatic cell volume and increase cellular deformation of the basaloid and sarcomatoid subtypes compared to the control group.

The rapid growth of cancer cells can exceed blood supply capacity, leading to areas of necrosis due to a lack of oxygen and nutrients.^85,86^ Compression of surrounding blood vessels can result in atrophy.^87^ Both cancer subtypes, basaloid and sarcomatoid, show high rates of mitosis and areas of necrosis, with basaloid characterized by comedonecrosis. Excessive mitotic activity concerning vascular and nutritional supply can result in tissue necrosis,^88,89^ influencing the area and volume measurements observed in AFM. Hypoxic conditions in the collagen-rich ECM, intensified by the interaction between cancer cells and collagen, affect vascular supply. Factors such as HIF-1, LOX, and metalloproteinase play roles in this process, as they are related to cancerous blood vessels. The firmness of collagen in the matrix affects vascular growth, impacting the formation of necrotic foci and fissures identified in AFM.^27^ The increase in deformation in the basaloid and sarcomatoid groups can be explained by the same mechanisms, given that the deformation is directly correlated with area and volume.

Raman spectroscopy analysis strongly corroborates these data. When a molecular group changes, the vibrational modes relative to it are also changed. Changes in the intensity, position, and broadening of the Raman spectrum peak can verify this fact. Significant differences between control and cancerous tissues are observed in the low-wavenumber range (700–1800 cm^–1^). These discrepancies involve vibrational modes related to proteins like proline (919 cm^–1^), which is abundant in collagen. Additionally, changes in the stretching of the C–N bonds and the stretching of the C=C quinoid ring (1392 and 1416 cm^–1^)^67,90^ indicate altered redox processes and compromised cellular metabolism.^91^ The range between 1220 and 1300 cm^–1^ is attributed to Amide III,^67,68^ and the region 1638 to 1665 cm^–1^ is attributed to Amide I,^67,68^ which are groups composed of carbon, oxygen, and nitrogen atoms (CONH) that play a crucial role in proteins formation. These bonds are essential for conferring structural rigidity and provide information about secondary structure organization in PCa tissues. The RS results showed that, in LWN, the vibrational modes of proteins in the basaloid subtype presented significantly higher intensities compared to the sarcomatoid subtype. In the basaloid subtype, this higher intensity is associated with the interactions of the HPV viral oncoproteins, E6 and E7, with cellular proteins, such as p53 and Rb, promoting structural and conformational changes that are captured in the Amide I, Amide III and proline bands. In contrast, in the sarcomatoid subtype, which is not related to HPV, the vibrational modes of the proteins are less intense, indicating less disturbance in the cellular pathways linked to these regulatory proteins.

Furthermore, changes were identified in the bands corresponding to lipids (1131 and 1381 cm^–1^). These modes reflect the composition, organization, and structure of lipids in penile cancer tissues, providing valuable information about the tissue’s biochemistry. The presence of cytosine (1514 cm^–1^) in PCa tissues indicates how mutations or epigenetic changes can be critical in transforming a normal cell into a cancerous cell.^92,93^

In the high wavenumber region (HWN) of the Raman spectrum, between 2700 and 3100 cm^–1^), stretch bands of the C–H bonds of lipids present in the membranes of PCa tissues are detected, described by Matthews et al.^94^ The peak at 2888 cm^–1^ is associated in the literature with the asymmetric stretching of lipids and proteins,^67^ which is generally observed in adipose,^95,96^ skin,^96,97^ and brain tissue.^96^ Despite not demonstrating the precursor of lipid breakdown, this result shows that these components are most likely related to carcinogenic transformation. These vibrations also provide crucial information about the composition and organization of lipids in the lipid layers of PCa tissues, playing a fundamental role in membrane integrity and permeability. In the same way, as in the low wavenumber region, we also observed variations in the intensities of the modes related to the biochemical groups associated with PCa. These results reinforce the potential of Raman Spectroscopy to distinguish penile cancer subtypes based on their molecular signatures and their different etiologies.

According to the principal component analysis (PCA) of the spectra, a statistically significant distinction can be observed between the spectra obtained in the control, sarcomatoid, and basaloid samples. This statistically substantial distinction indicates differences in the spectral characteristics of these groups. These differences can be explored to develop more accurate and efficient assessment methods for these types of tissues.

When performing an analysis of the spectral data of the basaloid and sarcomatoid subtypes, statistical significance was found, indicating the differences in the spectral characteristics. The fact that the ellipses are close together suggests a relationship or similarity between the basaloid and sarcomatoid subtypes. This fact means that although there are statistically significant differences in spectral characteristics, overlap or proximity exists in some areas of these subtypes. These results are important because they suggest exploring the similarities and differences identified to develop more accurate and efficient assessment methods to distinguish between control, basaloid, and sarcomatoid groups. In other words, multivariate analysis provides information about the global variation in spectral data. It highlights different and shared aspects between these groups, which can be useful in developing more refined assessment approaches.

## Conclusions

The study investigated the ultrastructural and vibrational properties of tissue subtypes of penile cancer (PCa) using Atomic Force Microscope (AFM) and Raman Spectroscopy (RS). AFM maps revealed ultrastructural changes in PCa tissues, indicating wear and stretching, possibly due to cytoskeleton and ECM reorganization that gradually promote cancer progression.^98^ Increased roughness of tissues affected by cancer suggests membrane erosion related to tumor invasion.^99^

Analysis of the surface area showed a reduction, especially in the basaloid subtype, associated with aggressiveness and high nodal metastasis. The decrease in basaloid and sarcomatoid sample volumes suggests possible necrosis, lack of blood supply, and cell volume regulation during exposure to osmotic stress. High rates of mitosis and areas of necrosis in these subtypes influence area and volume measurements. Hypoxic conditions in the collagen-rich extracellular matrix, related to factors such as HIF-1, LOX, and Metalloproteinase, affect vascular supply.

RS detected vibrational modes in PCa tissues, revealing spectral differences between control and cancerous samples. The discriminatory capacity of the techniques was confirmed by multivariate analysis, indicating significant differences between control and sarcomatoid/basaloid samples. PCA allowed the differentiation of the different groups, revealing distinct molecular patterns even when the spectral differences seemed subtle. The PCA distinction addresses one of the biggest challenges in studying penile cancer: distinguishing different tumor subtypes. In addition, RS data provided essential complementary information to AFM results. While AFM revealed changes in the morphological properties of the tissues, the Raman spectra allowed the identification of specific vibrational modes associated with biochemical changes, such as protein and lipid composition. By integrating Raman data with AFM findings, it is possible to build a more comprehensive profile of the tumor microenvironment, which has improved our understanding of ultrastructural and molecular alterations in cancerous tissues. These techniques offer a multifaceted approach to cancer characterization, providing structural and biochemical insights essential for advancing cancer diagnostics and treatment.

## Notes

The study received approval from the Research Ethics Committee on Human Subjects at the University Hospital of the Federal University of Maranhão (CEP/HU-UFMA) (CAAE 30760420.3.0000.5086) and was conducted by the Declaration of Helsinki. Considering the study’s retrospective nature, the Research Ethics Committee on Human Subjects at the University Hospital of the Federal University of Maranhão exempted the need for patient consent (Opinion Number 4.228.789).
